# Expression of ciliary neurotrophic factor (CNTF) and its tripartite receptor complex by cells of the human optic nerve head

**Published:** 2007-05-23

**Authors:** Xiaochun Liu, Abbot F. Clark, Robert J. Wordinger

**Affiliations:** 1 Department of Cell Biology and Genetics and The North Texas Eye Research Institute, University of North Texas Health Science Center at Fort Worth, TX; 2 Glaucoma Research, Alcon Research, Ltd., Fort Worth, TX

## Abstract

**Purpose:**

Ciliary neurotrophic factor (CNTF) promotes gene expression, cell survival and differentiation in various types of peripheral and central neurons, glia and nonneural cells. The level of CNTF rises rapidly upon injury to neural tissue, suggesting that CNTF exerts its cytoprotective effects after release from cells via mechanisms induced by cell injury. The purpose of this study was to determine if cells in the optic nerve head express CNTF and its tripartite receptor complex.

**Methods:**

Well-established optic nerve head astrocytes (ONHA) and lamina cribrosa (LC) cell cultures were derived from normal human donors. Total RNA was reverse transcribed and polymerase chain reaction (PCR) amplified for mRNA detection. Cytoplasmic protein expression was determined by immunocytochemistry and Western blot analysis of the cellular lysates. Serum free medium was concentrated and used for detecting extracellular proteins. CNTF complexed with CNTFR-α was assayed by immunoprecipitation.

**Results:**

Cells isolated from the human optic nerve head express CNTF and its tripartite receptor complex members (CNTFR-α, gp130, LIFR-β).

**Conclusions:**

Taken together, these data suggest a possible neuroprotective role of CNTF in the optic nerve head.

## Introduction

Primary open angle glaucoma (POAG) affects about 70 million people worldwide with the characteristic optic neuropathy of retinal ganglion cell death and axon loss, which reflects morphologically as optic nerve head (ONH) cupping. Despite vigorous research efforts, current treatments do little to reverse the course of visual loss.

Within the human ONH, the lamina cribrosa (LC) region physically protects nerve axon fibers from structural distortion under stress. The LC region is composed of glial cell columns and connective tissue plates that form channels to guide and support retinal ganglion cell axons as they exit the eye. The glial and support cells in this region normally express trophic factors including neurotrophins [1], suggesting that growth factors and cytokines may provide nerve protection at the molecular level. We set out to study the expression of ciliary neurotrophic factor and its receptor complex in the cells of the LC region.

Among various neurotrophic factors, ciliary neurotrophic factor (CNTF) is an injury-induced trophic factor that provides protection for multiple types of neurons (e.g. sensory, sympathetic, motor neurons) and glial cell populations [2], possibly through STAT3 activation [3]. CNTF is a member of the α-helical neuropoietic cytokine family that also includes leukemia inhibiting factor (LIF), oncostatin M (OSM), interleukin-6 (IL-6), interleukin-11 (IL-11), cardiotrophin-1 (CT-1), cardiotrophin-like cytokine/cytokine-like factor-1 (CLC/CLF, or CNTF-2) [4,5], and neuropoietin [6]. Originally identified as a survival factor for chick ciliary ganglion neurons [7,8], exogenous CNTF both enhances expansion of the number of stem cells in the adult forebrain in vivo [9] and stimulates cell differentiation in cultures of retinas and sympathetic neurons [10-12]. In the eye, CNTF promotes axonal genesis in dissociated retinal ganglion cells [13], and axotomized retinal ganglion cells in adult hamsters [14] and rodents [15]. Many pathologic conditions including ischemia can induce the levels of CNTF in vivo. Under those circumstances, CNTF may be a regulator of gliogenesis or gliosis, a reactive process of the glial cells following different types of insults such as ischemia, infection or malformation in the nervous system [16-18]. In mouse retina, intravitreal injection of CNTF can increase GFAP promoter activity in Müller cells [19], which is consistent with the presence of a CNTF-responsive element in the GFAP promoter [20]. Altogether, CNTF appears to be a pleiotropic cytokine that provides support against neuronal degeneration following insult as well as trauma.

CNTF signals through its tripartite receptor complex consisting of a α-receptor (CNTFR-α), a β-receptor (Leukemia inhibitory factor receptor, LIFR-β) and a glycoprotein (gp130). CNTFR-α is distinct from other neurotrophic or neurotrophin receptors in that it is anchored to the cell membrane via a glycosylphophatidylinositol (GPI) linkage. Upon binding to CNTF, CNTFR-α triggers the formation of a heterodimer between LIFR-β complex, and gp130 [18], which activates downstream signaling molecules. In this study, we demonstrated that two ONH cell types (i.e. ONH astrocytes and LC cells [21]) express mRNA and protein for CNTF and its tripartite receptor complex (CNTFR-α, gp130, LIFRβ).

## Methods

### Cell culture

Human optic nerve head astrocytes (ONHA) and LC cells were isolated and characterized as described previously [1,22]. LC cells (from 8-month, 36-weeks, 66-, 89-, and 90-year donors) were maintained in Ham's F-10 Media (JRH Biosciences, Lenexa, KS) supplemented with 10% FBS, L-glutamine (0.292 mg/ml), penicillin (100 units/ml)/streptomycin (0.1 mg/ml), and amphotericin B (4 mg/ml). ONHA (from 36-week, 89-, and 90-year donors) and normal human brain astrocytes (NHA; Clonetics, San Diego, CA) were maintained in AGM (Clonetics, San Diego, CA) containing 5% fetal bovine serum (FBS), human epidermal growth factor (EGF), insulin, progesterone, transferrin, gentamicin, and amphotericin-B. Brain astrocytes were used as a positive control for the mRNA expression of CNTF and the CNTF receptor complex. All cell lines were maintained at 37 °C in 5% CO_2_-95% room air, and medium was changed every 2 to 3 days [21]. Normal human ONH tissue samples were pooled from normal donors of 76- to 97-years of age.

### Total cellular RNA extraction and cDNA synthesis

Total cellular RNA from 1x10^7^ cells was prepared using the TRIzol reagent (Invitrogen Life Technologies, Carlsbad, CA). After isopropanol precipitation, the RNA was resuspended in 20 μl of water and stored at -80 °C. First strand complementary DNA (cDNA) synthesis from total cellular and tissue RNA and details of PCR procedure were prepared as previously described [23].

### Immunocytochemical localization of ciliary neurotrophic factor and its receptor complex

Immunocytochemistry studies of CNTF and its receptor complex in LC cell lines and ONHA were performed as previously described [23]. Fluorescence was detected using a Nikon Microphot-FXA microscope with the appropriate filter. Images were recorded and processed using IPLabs 5.0 software (Scanalysis, Inc., Fairfax, VA). Control immunohistochemical preparations included both (a) omission of the primary antibody and (b) neutralization of the primary antibody with a 10-fold (by weight) excess of blocking peptide (Santa Cruz Biotechnology, Inc., Santa Cruz, CA) in PBS overnight at 4 °C.

### Western blot analysis of ciliary neurotrophic factor and Its receptor complex

Proteins from confluent or near confluent LC cells, and ONHA (1x10^7^) were extracted and assayed via Western blot analysis as previously reported [23]. Omission of the primary antibodies served as negative controls. Western blots were repeated twice to confirm results. After the final wash, membranes were treated with enhanced chemiluminescence (ECL) Western blotting kit detection reagents (Amersham Pharmacia Biotech, Piscataway, NJ) and exposed to Hyperfilm ECL (Amersham Pharmacia Biotech) for the period of time ranging from 1 s to 30 min depending on the amount of target protein. Some of the images were also captured using the computerized charge-coupled device (CCD) camera-based imaging system (Alpha Innotech, San Leadro, CA) following application of SuperSignal West Femto Maximum Sensitivity Substrate (Pierce Biotechnology, Inc. Rockford, IL) on the membranes.

### Immunoprecipitation

Human ONHA and LC cells (1x10^7^ cells) were grown in serum-free culture medium for 48 h. The serum-free medium was collected and concentrated 20-fold using centricon YM-3 (Millipore Corporate, Billerica, MA). Cellular protein was collected in lysis buffer (150 mM NaCl, 1% Triton X-100, 50 mM Tris). A protease inhibitor cocktail (Sigma-Aldrich, St. Louis, MO) was added to each sample upon collection. Protein concentration was measured using the Bio-Rad Dc Protein Assay System (Bio-Rad Laboratories, Richmond, CA). Cellular lysate (100 μg) was incubated with 5 μg goat anti-CNTFR-α IgG overnight. Samples were then incubated with 50 μl of 50% protein G-agarose for 2 h at 4 °C, washed 4 times, and resuspended in sample buffer. Immunoprecipitated proteins were separated on 10% denaturing polyacrylamide gels and electrophoretically transferred to nitrocellulose membranes. Blots were blocked and analyzed as described in Western blot analysis section above.

## Results

### Expression of ciliary neurotrophic factor and ciliary neurotrophic factor tripartite receptor complex mRNAs in human lamina cribrosa cells and optic nerve head astrocytes

**Figure 1:**
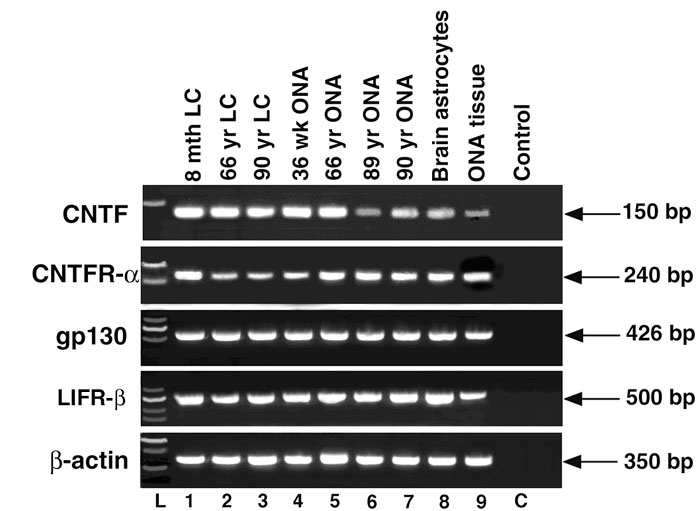
Ethidium bromide-stained agarose gel of ciliary neurotrophic factor and the ciliary neurotrophic factor tripartite receptor complex polymerase chain reaction products from optic nerve head cells. Lamina cribrosa cells are lanes 1-3, optic nerve head astrocytes (ONHA) are lanes 4-7, brain astrocytes are lane 8, and optic nerve head tissue (ONA tissue) is lane 9. C indicates negative control (i.e. no template reaction control). L indicates molecular size ladder. Each lane corresponds to an individual donor. Lamina cribrosa (LC) cells are from donors of 8 month old (8 mth LC), 66 year old (66 yr LC), and 90 year old (90 yr LC). ONHA cells are from donors of 36 week old (36 wk ONA), 66 year old (66 yr ONA), 89 year old (89 yr ONA), 90 year old (90 yr ONA). β-actin (350 bp) was included to monitor potential genomic DNA contamination.

The mRNA expression of CNTF, CNTFR-α, gp130, and LIFR-β from LC cells and ONHA are represented as PCR products in Figure 1. In all cell lines tested, including a brain astrocyte cell line that is known to express CNTF and its receptor complex, a single band at the correct size was detected following gel electrophoresis. This PCR product is unlikely a genomic DNA contamination, as a set of β-actin primers that span an intron did not amplify a genomic fragment. Additionally, in an optic nerve head tissue sample (Lane 9), the presence of a PCR product confirmed the in vivo expression of CNTF and the receptor complex. Therefore, human LC cells and ONHA express mRNA for CNTF and its tripartite receptor complex (i.e. CNTFR-α, gp130 and LIFR-β).

### Expression of ciliary neurotrophic factor and the ciliary neurotrophic factor tripartite receptor complex proteins in human lamina cribrosa cells and optic nerve head astrocytes

**Figure 2:**
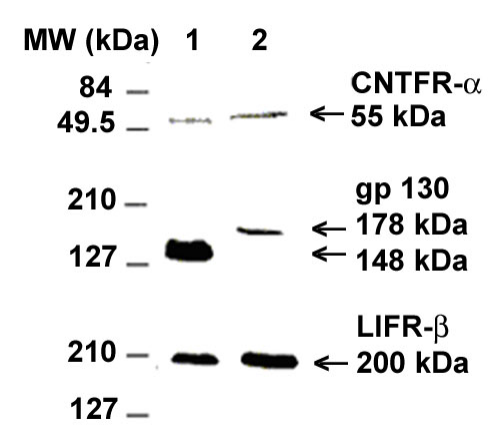
Chemiluminescent detection of the ciliary neurotrophic factor tripartite receptor complex in optic nerve head cells. Western immunoblot of lamina cribrosa cell lysate (Lane 1) and optic nerve head astrocytes lysate (Lane 2).

Figure 2 demonstrates the protein expression of CNTFR-α, gp130, and LIFR-β in human LC cells and ONHA by the Western blot analysis. For each protein, the specific antibody recognized a single band from both cell types. Without the primary antibody, no band was recognized on a gel that was run and analyzed in parallel, which ruled out the nonspecific reactivity of the secondary antibody. In agreement with the literature [24,25], we detected a 52.3 kDa protein for CNTFR-α and a 190-210 kDa protein for LIFR-β in both cell types. Gp130 stained as a canonical 130-150 kDa protein in LC cells [24,26]. Despite our numerous attempts, we were unable to detect CNTF protein in either LC cells or ONHA cell lysate (data not shown).

**Figure 3:**
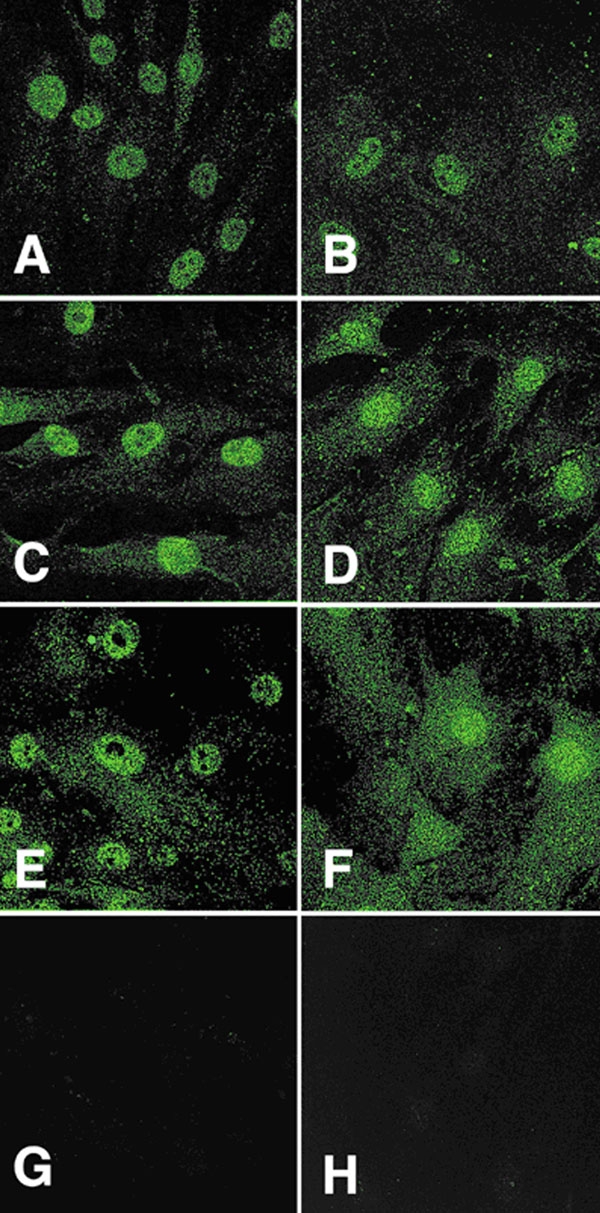
Immunofluorescent localization of ciliary neurotrophic factor tripartite receptor complex proteins in optic nerve head cells. Lamina cribrosa cells are in **A**, **C**, and **E** and optic nerve head astrocytes are in plates **B**, **D**, and **F**. **A** and **B** are examples of ciliary neurotrophic factor-α staining of the cells; plates **C** and **D** correspond to LIFR-β cellular staining; **E** and **F** are representatives of gp130 staining of both cell types; **G** and **H** are examples of negative controls (primary antibody omitted).

Complementary to the Western blot analysis, our immunocytochemistry study localized CNTFR-α, gp130, and LIFR-β proteins in both LC cells and ONHA from multiple donors in vitro. A representative picture of each receptor component in each cell type is shown in Figure 3. All members of the CNTF receptor complex were localized in both cell types. The staining pattern for all three components appeared punctate, resembling typical membrane proteins. There were no obvious differences in either intensity or staining pattern between LC cells and ONHA. When primary antibodies were omitted or blocking peptide neutralization was implemented, no staining was observed. There was no detectable fluorescent signal for CNTF in either cell type (data not shown). This is in agreement with previous literature that CNTF is detectable only at very low levels in the intact adult central nervous system (CNS).

### Ciliary neurotrophic factor-ciliary neurotrophic factor receptor-α complex protein expression by lamina cribrosa cells and optic nerve head astrocytes

**Figure 4:**
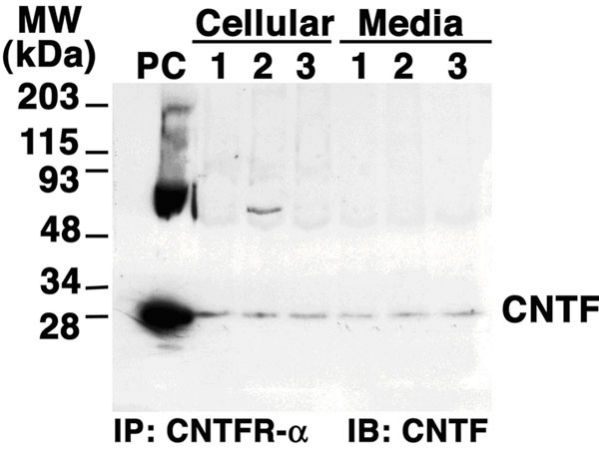
Anti-ciliary neurotrophic factor western immunoblots of ciliary neurotrophic factor receptor-α immunoprecipitated proteins from lamina cribrosa cell and optic nerve head astrocyte lysates and cell culture medium. PC indicates positive control (recombinant ciliary neurotrophic factor protein). Lane 1 represents LC cells from a 90 year old donor; lane 2 represents optic nerve head astrocyte cells from a 66 year old donor; lane 3 represents ONHA cells from a 90 year old donor.

A CNTF-CNTFR-α protein complex was detected in LC cells and ONHA, although expression of CNTF was at extremely low levels. The cell lysates and the serum-free culture medium of both cell lines were concentrated and subjected to immunoprecipitation with a specific antibody against CNTFR-α. The resultant mixture was then enriched with CNTFR-α and its complexes. CNTF-CNTFR-α complexes in all samples were revealed by western immunoblotting using a specific antibody against CNTF (Figure 4). From both cell lysates and conditioned media, we were able to detect a single CNTF protein band of appropriate molecular weight. Our results detected the expression of CNTF protein in complex with CNTFR-α in both LC cells and ONHA. The necessity of extensive concentration procedures supported the previous notion that CNTF protein expression is normally extremely low.

## Discussion

Our study provides direct evidence that cultured LC cells and ONHA constitutively express CNTF and its tripartite receptor complex. The presence of the CNTF receptor complex enables both LC cells and ONHA to respond to CNTF. The ONH harbors LC cells and ONHA, which can be potential sources of CNTF during stressful events [1,10,21,27]. Therefore, CNTF can signal ONH cells in a paracrine as well as autocrine fashion.

Consistent with previous reports in the brain, CNTF is expressed at low levels in these ONH cell types under normal conditions. As a member of the IL-6 growth factor family, CNTF shares some of the receptor components with the family members in forming a multi-component signal-transducing receptor complex. Presumably, there are overlapping biological activities and a degree of functional redundancy between CNTF and other family members. Conceivably, IL-6 family members other than CNTF may maintain the physiological function of the ONH through the receptors under normal conditions. Our study raises the possibility of a neuroprotective role of CNTF in human optic nerve head via a paracrine/autocrine mechanism.

Since CNTF does not contain a classical signal sequence for secretion, it has long been postulated that CNTF are released by cytolysis due to injury. A recent study of bovine corneal epithelial cells [28] suggested that CNTF may be externalized while binding to CNTFR-α. As a glycosyl-phosphatidylinositol (GPI)-anchored coreceptor, CNTFR-α can carry CNTF to the outer leaflets of the cell membrane and be released to the extracellular space upon phospholipase cleavage. This theory is not completely novel, as cardiotropin-like cytokine (CLC), a member of the CNTF neurotrophic factor family, generates a similar functional composite cytokine with CNTFR-α (CLC-CNTFR-α complex) in mammalian cells [29]. Our results support this type of mechanism. First of all, the immunoprecipitation study demonstrated that CNTF-CNTFR-α complex is present extracellularly, indicating that CNTF and its α receptor are released as a complex. Secondly, under normal conditions, there is minimal cytolysis, and the CNTF released by disintegration of cells is below the threshold of detection. Therefore, the presence of CNTF in the cell culture medium is more than likely due to externalization and release of the CNTF-CNTFR complex instead of cytolysis.

In conclusion, our study demonstrates the potential for a significant signaling system using CNTF family neurotrophic factors in glial cells of the optic nerve head. In addition to the current study, we have also reported that neurotrophin signaling systems in LC cells, ONHA, and ONH tissue [1]. We have also reported the presence of glial-derived neurotrophic factor (GDNF) and the receptor complex in the same cell types [27]. Neurotrophic factors and neurotrophins may play a critical role in restoring the normal function of lamina cribrosa, especially during the time of injury. This implies that neurotrophin and neurotrophic factors may have therapeutic applications for neuroprotection in glaucoma patients.
